# Outcome measurement in blue space and depression-related research: a systematic review and exploratory methodological evidence synthesis

**DOI:** 10.3389/fpsyg.2026.1891489

**Published:** 2026-08-03

**Authors:** Boyi Yang, Yanyan Zhou, Aichun Li, Wuhu Xu

**Affiliations:** 1 School of Physical Education, Hainan Normal University, Haikou, China; 2 School of Agriculture and Environment, University of Western Australia, Perth, WA, Australia

**Keywords:** blue space, evidence synthesis, mental health, outcome measurement, psychological distress, psychometrics, systematic review

## Abstract

Blue-space exposure has been studied in relation to mental health, but studies have often labeled non-equivalent outcomes—including depression-focused symptoms, psychological distress, mental-health functioning, diagnostic disorders, and binary self-reports—as “depression.” This systematic review examined how depression-related outcomes were operationalized in studies of adult blue-space exposure and whether the available evidence supported quantitative synthesis. The review was reported in accordance with the PRISMA 2020 reporting guideline. Six databases were searched for studies published between January 2000 and March 2026, and the protocol was prospectively registered in PROSPERO. Eight observational studies involving 35,797 adults were included. Blue-space exposure definitions, exposure contrasts, outcome constructs, covariate adjustment, and effect metrics varied substantially. One study using the 15-item Geriatric Depression Scale reported an inverse adjusted association between street-view blue space and geriatric depressive symptoms, but its exposure contrast, outcome scale, population, and setting were not directly comparable with those of the other studies. Because the available estimates could not be transparently expressed on a common effect scale, no meta-analysis was performed; findings were synthesized as structured study-level evidence. Current evidence does not establish whether associations between blue-space exposure and depression-related outcomes differ by outcome-measurement category. Future studies should prespecify the target construct, select measures aligned with the proposed mechanism and study population, and report sufficient domain- or item-level information to support transparent evidence synthesis.

## 1 Introduction

Blue spaces, including rivers, lakes, coastlines, seas, and other outdoor water bodies, have received increasing attention as potentially health-supportive environmental resources. Alongside individual-level risk factors, environmental conditions across the life course may shape mental health through pathways involving restoration, stress reduction, physical activity, social interaction, place attachment, and affective regulation (White et al., 2020; Smith et al., 2021; Wang et al., 2025). Empirical studies have therefore examined whether residential proximity to water, visible blue space, coastal distance, water coverage around the home, and other forms of blue-space exposure are associated with depression-related outcomes (Helbich et al., 2019; White et al., 2013; Liu et al., 2020, 2021; Chen and Yuan, 2020; Nutsford et al., 2016; Triguero-Mas et al., 2015; de Vries et al., 2016). Previous reviews have generally suggested potentially favorable associations between natural environments and mental-health outcomes, while also emphasizing substantial variation in exposure definitions, study populations, research designs, and outcome measures (Smith et al., 2021; Liu et al., 2023; Wang et al., 2025). However, residential environmental exposure studies, visibility-based studies, studies of actual blue-space use, and nature-based or green-exercise interventions represent different exposure constructs and causal contrasts. Consequently, variation in observed associations may reflect not only differences in mental-health measurement, but also differences in environmental context, exposure intensity, accessibility, surrounding neighborhood conditions, population characteristics, and the social or psychological pathways through which blue space may influence mental health. Depressive disorders remain a major contributor to the global burden of mental disorders (GBD 2019 Mental Disorders Collaborators, 2022).

Within blue-space research, outcome measurement is one important but underexamined source of interpretive uncertainty. Studies have used the Geriatric Depression Scale-15 (GDS-15), General Health Questionnaire-12 (GHQ-12), Mental Health Inventory-5 (MHI-5), SF-36 Mental Health subscale, Kessler Psychological Distress Scale (K10), Composite International Diagnostic Interview (CIDI), and binary self-reported depression or anxiety items. These instruments differ in their format, intended use, item content, scoring direction, and clinical purpose. Such diversity is not inherently problematic. Different measures may be appropriate for different populations, research questions, and hypothesized pathways linking blue-space exposure to mental health. For example, a depression-focused symptom measure may be appropriate when the research question concerns depressive symptom burden, whereas a broad distress or mental-health-functioning measure may be appropriate when the proposed pathway concerns stress recovery, emotional wellbeing, or general psychological functioning. Similarly, structured diagnostic interviews and binary self-reports address different levels of mental-health assessment and may be appropriate for different study objectives. The interpretive concern arises only when measures with different construct coverage are treated as direct equivalents without clearly specifying what each instrument captures.

This issue is particularly relevant because depression-related outcomes are not unitary. Depression may involve affective, cognitive, somatic, motivational, interpersonal, and functional components, and commonly used scales differ substantially in symptom content and construct emphasis (Fried and Nesse, 2015a; Fried, 2017; Maj et al., 2020). The GDS-15 was developed as a depression screening instrument for older adults and reduces the influence of somatic symptoms that may overlap with aging and physical illness (Yesavage et al., 1982; D’Ath et al., 1994; Zhang et al., 2022). The GHQ-12 and K10 primarily assess broad psychological distress or nonspecific psychological morbidity (Goldberg and Hillier, 1979; Kessler et al., 2002; Hystad and Johnsen, 2020; Anjara et al., 2020). By contrast, the MHI-5 and SF-36 Mental Health subscale assess broader emotional well-being and mental-health functioning (Ware and Sherbourne, 1992; Bech et al., 2003; Friedman et al., 2005; Yamazaki et al., 2005; Ten Have et al., 2024). These measures are clinically meaningful and may be relevant to depression-related research, but they differ in specificity, item content, diagnostic purpose, and sensitivity to particular symptom domains. Therefore, when findings are compared across studies, the meaning of an estimated association depends not only on the blue-space exposure being measured but also on the psychological construct represented by the outcome measure.

The present study was designed as a systematic review with an exploratory methodological evidence synthesis of outcome measurement in studies examining adult blue-space exposure and depression-related outcomes. Rather than determining whether one measurement category is psychometrically superior to another, or whether outcome measurement causally modifies the association between blue space and mental health, we aimed to describe the constructs represented by the included outcome measures and assess whether the available evidence was sufficiently construct-explicit and comparable to support quantitative synthesis. We classified outcomes as depression-focused symptom measures, broad psychological distress measures, mental-health-functioning measures, structured diagnostic outcomes, or binary self-reported outcomes. We also examined whether outcome-measurement categories co-occurred with other study-level characteristics, including population age, geographical setting, exposure assessment method, study design, and covariate structure. By doing so, this review aims to clarify what can and cannot currently be inferred from the blue space–depression-related evidence base and to identify priorities for more theory-aligned, construct-transparent future research.

## 2 Materials and methods

### 2.1 Study design and protocol

We conducted a systematic review with an exploratory methodological evidence synthesis of how depression-related outcomes were operationalized in studies of adult blue space exposure. The review was reported in accordance with the Preferred Reporting Items for Systematic Reviews and Meta-Analyses (PRISMA 2020) reporting guideline (Page et al., 2021). PRISMA was used to support transparent reporting of study identification, screening, eligibility assessment, data extraction, and evidence synthesis; it was not used as a methodological framework for conducting the review.

The exploratory component was designed to map the constructs represented by the included outcome measures and to evaluate the comparability of available study estimates. It was not intended to establish causal moderation by outcome-measurement category or to identify a universally preferred instrument. The review protocol was prospectively registered in the International Prospective Register of Systematic Reviews (PROSPERO; CRD420261340412) before title and abstract screening and data extraction commenced.

### 2.2 Data sources and search strategy

Database searches were conducted in Web of Science, PubMed, Scopus, APA PsycInfo, China National Knowledge Infrastructure (CNKI), and Wanfang. The search window covered publications from 1 January 2000 to 31 March 2026. Search terms combined blue space-related keywords with adult population terms and depression-related outcomes. English terms included “blue space,” “river,” “lake,” “sea,” “coastal,” “adult,” “depression,” and “depressive symptoms.” Chinese terms included “蓝色空间,” “江,” “河,” “湖,” “海,” “滨水,” “海岸,” “成年人,” “居民,” “抑郁,” and “抑郁症状.” The full search strategy is reported in Table 1. The complete database-specific search strategies, including field tags, search limits, search dates, and the number of records identified from each database, are provided in Supplementary Table 1.

**TABLE 1:** Core search concepts and example PubMed search strategy.

Database	Search string
Chinese (CNKI/Wanfang)	(蓝色空间 OR 江 OR 河 OR 湖 OR 海 OR 滨水 OR 海岸 OR 湿地) AND (成年人 OR 居民 OR 青年 OR 中年) AND (抑郁 OR 抑郁症状 OR 抑郁症)
English PubMed (example search strategy)	(“blue space”[Title/Abstract] OR “river”[Title/Abstract] OR “lake”[Title/Abstract] OR “sea”[Title/Abstract] OR “coastal”[Title/Abstract]) AND (“adult”[MeSH] OR “adult”[Title/Abstract]) AND (“depression”[MeSH] OR “depressive symptoms”[Title/Abstract])

Database-specific search strings, platform-specific field tags, filters, and search dates for Web of Science, Scopus, APA PsycInfo, CNKI, and Wanfang are reported in Supplementary Table 1.

### 2.3 Eligibility criteria

Eligibility was defined using the PICOS framework. Eligible studies enrolled adults aged 18 years or older, assessed exposure to outdoor blue spaces such as rivers, lakes, coastlines, seas, or other natural water bodies, and reported at least one of the following outcomes: (a) depressive symptoms assessed using a depression-focused questionnaire; (b) broad psychological distress or mental-health functioning assessed using a validated questionnaire; (c) a structured diagnostic mood-disorder outcome; or (d) a binary self-reported depression or anxiety outcome. In this review, these outcomes were collectively termed “depression-related outcomes” for descriptive purposes only and were not assumed to be construct-equivalent. Cross-sectional, cohort, and case-control designs were considered eligible when the full text was available in English or Chinese.

Studies were excluded when they examined green space only without separately reporting blue space effects, reported combined green-blue exposure effects that could not be disentangled, focused only on artificial or indoor water features, assessed general wellbeing without depression-related items, or were reviews, conference abstracts, editorials, animal studies, or duplicate publications.

### 2.4 Data extraction

Two reviewers independently extracted study characteristics and analytic information from each included study, including first author, publication year, country, study design, sample size, mean age, blue space exposure metric, depression-related outcome instrument, effect estimate, confidence interval, and covariates adjusted for in the original analysis. Particular attention was given to the outcome instrument used to assess depressive symptoms or depression-related distress, because this variable formed the basis of the outcome-measurement audit.

The two reviewers cross-checked the extracted data, and disagreements were resolved through discussion. When a study reported multiple eligible estimates, one estimate was selected using a predefined hierarchy. First, we selected the primary blue-space exposure identified by the original authors. Second, for that exposure, we selected the adjusted model identified by the original authors as their primary or fully adjusted model. Third, we selected estimates from the full analytic sample rather than subgroup-specific estimates.

When a study reported several exposure metrics but did not identify a primary exposure, and the metrics represented non-equivalent constructs such as visibility, proximity, coverage, or use, we did not select an estimate on the basis of effect magnitude or statistical significance. Instead, the study was retained in the structured study-level synthesis unless a transparent, prespecified rationale supported the descriptive presentation of one estimate. All eligible alternative estimates, exposure buffers, original models, and estimate-selection decisions are reported in Supplementary Table 3. The characteristics of the included studies, original exposure contrasts, selected adjusted estimates, and estimate-selection decisions are summarized in Table 2.

**TABLE 2:** Characteristics of included studies, exposure contrasts, outcome constructs, and adjusted estimates.

No.	Study, country, and design	Sample	Outcome measure and review classification	Blue-space exposure construct and original contrast	Original adjusted effect estimate (95% CI)	Selected model and key covariates	Estimate-selection rationale	Synthesis role
1	Helbich et al. (2019), China; cross-sectional	*n* = 1,190; mean age = 70.7 years	GDS-15; depression-focused geriatric symptom measure	Street-view visible blue-space proportion; Q2–Q4 versus Q1	For Q4 versus Q1: β = −1.059 (−1.951 to −0.167)	Multilevel linear regression with neighborhood random intercept; fully adjusted street-view model jointly included street-view green and blue space. Covariates: age, gender, education, ethnicity, marital status, party membership, hukou status, functional ability, chronic disease status, and NO_2_.	All Q2–Q4 versus Q1 estimates were retained in Supplementary Table 3. The Q4-versus-Q1 estimate is displayed descriptively because no single quartile contrast was designated as the primary exposure contrast.	Structured study-level evidence; no pooled effect
2	White et al. (2013), United Kingdom; longitudinal panel study	*n* = 15,361; mean age ≈ 45 years	GHQ-12 inverse mental-health score; broad psychological distress	Coastal proximity; 0–5 km from the coast versus 45–50 km from the coast (reference)	B = 0.147 (0.017 to 0.277)^†^	Individual fixed-effects linear panel model. Area-level variables: green-space coverage, freshwater coverage, area income, employment, education, and crime. Individual-level variables: age, education, marital status, children in household, household income, work-limiting health, employment status, housing type, household space, and commuting time.	Coastal-distance categories represented a non-linear exposure gradient. The 0–5 km versus 45–50 km reference-category contrast was retained because it was explicitly reported in the original fixed-effects model. All eligible coastal-distance estimates are reported in Supplementary Table 3.	Structured study-level evidence; no pooled effect
3	Liu et al. (2021), China; cross-sectional	*n* = 1,274; mean age ≈ 40 years	MHI-5; mental-health-functioning measure	Accessible water area within 300 m; per 1-hectare increase	β = 0.325 (0.033 to 0.617)^†^	Fully adjusted linear regression model. Covariates included age, gender, education, income, occupation, marital status, residential location/urbanicity, and recent life events.	The per-hectare water-area estimate was retained because it represented the reported blue-space association in the fully adjusted model. It was not converted to a common SMD because its exposure unit was not comparable with visibility, categorical proximity, or index-based contrasts.	Structured study-level evidence; no pooled effect
4	Chen and Yuan (2020), China; cross-sectional	*n* = 966; mean age ≈ 70 years	SF-36 Mental Health subscale; mental-health-functioning measure	Hydrophilicity index; index value 1 versus 0	β = 0.744 (−0.060 to 1.548)^†^	Fully adjusted multilevel linear model. Covariates: age, gender, education, marital status, hukou status, household income, and occupational status. Potential mediators were analyzed separately and were not treated as routine confounders.	Several non-equivalent blue-space metrics were reported. The hydrophilicity index was retained to preserve the original extraction decision; alternative water amount, proximity, and accessibility indicators are documented in Supplementary Table 3.	Favourable direction; 95% CI included the null
5	Nutsford et al. (2016), New Zealand; cross-sectional	*n* = 442; mean age ≈ 45 years	K10; broad psychological distress measure	Total visible blue space; per 10-percentage-point increase	β = −0.280 (−0.410 to −0.150)	Cluster-robust linear regression using imputed data. Covariates: sex, age, household income, neighborhood deprivation, neighborhood housing quality, population density, and crime rate; complex sampling design was accounted for.	Visible blue space was the authors’ focal blue-space exposure. The adjusted per-10% estimate was retained as descriptive study-level evidence; it was not treated as exchangeable with other exposure units.	Structured study-level evidence; no pooled effect
6	Liu et al. (2020), China; cross-sectional	*n* = 1,150; mean age = 39.6 years	GHQ-12; broad psychological distress measure	Street-view blue-space ratio within 1,500 m; per 1% increase	β = −0.406 (−0.785 to −0.027)	Multilevel structural equation model. Covariates: gender, age, educational attainment, marital status, hukou status, employment status, medical-insurance participation, and annual household income per household member. Neighborhood attachment, neighborly interaction, and community participation were modeled as mediators.	The study compared street-view and remote-sensing blue-space measures. The street-view direct path is shown for descriptive transparency, while both blue-space metrics are retained in Supplementary Table 3; neither was selected for pooled analysis.	Structured study-level evidence; no pooled effect
7	Triguero-Mas et al. (2015), Spain; cross-sectional	*n* = 8,793; mean age ≈ 41 years	Self-reported depression/anxiety; binary self-reported emotional problem	Presence of blue space within 300 m; yes versus no	OR = 1.13 (0.90 to 1.41)	Adjusted logistic regression in the full sample. Reported adjustment domains included physical activity, gender, urbanicity, socioeconomic status, and social support.	The full-sample adjusted estimate for blue space within 300 m was retained. The binary self-reported outcome and binary exposure contrast were not comparable with the continuous-outcome studies.	Narrative evidence only
8	de Vries et al. (2016), Netherlands; cross-sectional	*n* = 6,621; mean age ≈ 45 years	CIDI mood-disorder outcome; structured diagnostic mood disorder	Blue-space availability within 1 km; per 1-percentage-point increase	OR = 0.97 (0.951 to 0.989)	Multilevel logistic regression, Model 3. Covariates: gender, age, partner status, children in the household, educational level, paid employment, household income, green-space availability, and urbanicity. Property-value-adjusted models were sensitivity analyses.	Model 3 was retained because it represented the main jointly adjusted environmental model for the CIDI mood-disorder outcome. Alternative models and outcomes are documented in Supplementary Table 3.	Narrative evidence only

Effects are the original studies’ adjusted estimates and are reported in their original exposure units; they were not converted into standardized mean differences. A negative β indicates lower symptom burden or psychological distress for GDS-15, K10, and the GHQ-12 model reported by Liu et al. (2020). Positive coefficients for the inverse GHQ-12 score in White et al. (2013), MHI-5, and the SF-36 Mental Health subscale indicate better mental-health status. For binary outcomes, OR < 1 indicates lower odds of the reported outcome. β, regression coefficient; CI, confidence interval; CIDI, Composite International Diagnostic Interview; GDS-15, 15-item Geriatric Depression Scale; GHQ-12, 12-item General Health Questionnaire; K10, Kessler Psychological Distress Scale; MHI-5, 5-item Mental Health Inventory; NO_2_, nitrogen dioxide; OR, odds ratio; SF-36, 36-item Short-Form Health Survey.

† The coefficient direction was interpreted according to the original outcome-scoring system. Positive coefficients indicate better mental-health functioning or lower psychological distress when the outcome was inversely scored in the original study.

### 2.5 Quality assessment

Methodological quality was assessed with the Downs and Black checklist (Downs and Black, 1998). The assessment covered study design, confounding control, exposure measurement, outcome measurement, results reporting, statistical testing, sample representativeness, and follow-up duration. Each domain was scored from 0 to 2, yielding a maximum total score of 16. Scores ≥13 were classified as excellent, 10–12 as good, 7–9 as fair, and ≤6 as poor. Quality scores were used to describe study limitations and inform interpretation rather than to determine study eligibility.

### 2.6 Outcome-measurement coding framework

Given the heterogeneity of instruments used across the included studies, we conducted an exploratory outcome-measurement classification. Each instrument was coded according to its original measurement purpose, dominant item content, intended use, and degree of depression specificity. The classification was designed to support construct-transparent interpretation of study findings; it was not intended to rank instruments by validity or to imply that each instrument measured only one psychological construct.

Outcomes were classified into four descriptive categories: (1) depression-focused symptom measures; (2) broad psychological distress measures; (3) mental-health-functioning measures; and (4) structured diagnostic or binary self-reported outcomes. The GDS-15 was classified as a depression-focused geriatric symptom measure because it was developed to screen for depressive symptoms in older adults while reducing the influence of somatic symptoms that may overlap with aging and physical illness (Yesavage et al., 1982; D’Ath et al., 1994; Zhang et al., 2022).

The GHQ-12 and K10 were classified as broad psychological distress measures because they were developed to assess non-specific psychological morbidity or distress rather than depression-specific symptom severity (Goldberg and Hillier, 1979; Kessler et al., 2002; Hystad and Johnsen, 2020; Anjara et al., 2020). The MHI-5 and SF-36 Mental Health subscale were classified as mental-health-functioning measures because they assess broad emotional wellbeing and mental-health status; these instruments may be clinically informative and may function as screens for common mood or anxiety disorders in some settings, but they do not provide the same construct-specific information as depression-focused symptom scales (Ware and Sherbourne, 1992; Friedman et al., 2005; Ten Have et al., 2024).

The CIDI mood-disorder outcome was classified as a structured diagnostic outcome, whereas single-item self-reported depression/anxiety was classified as a binary self-reported outcome. Because these outcomes differ in ascertainment, scale, and clinical interpretation, they were not pooled with continuous symptom or distress measures (Kessler and Üstün, 2004; Haro et al., 2006). Two reviewers independently coded all instruments, and disagreements were resolved through discussion with a third reviewer. The interpretive classification of the outcome instruments is summarized in Table 3.

**TABLE 3:** Interpretive classification of depression-related outcome measures.

Instrument	Original intended construct	Interpretive construct coverage	Dominant content/domain	Review classification	Synthesis role
GDS-15	Geriatric depressive symptoms	Depression-focused construct coverage	Geriatric depressive symptom content, with reduced somatic emphasis	Depression-focused geriatric symptom measure	Structured study-level synthesis
GHQ-12	General psychological distress	Broad construct coverage	Broad non-specific psychological distress	Broad psychological distress measure	Structured study-level synthesis
MHI-5	Mental health status	Broad construct coverage	Emotional wellbeing and mental-health functioning	Mental-health-functioning measure	Structured study-level synthesis
SF-36 MH	Mental health functioning	Broad construct coverage	Emotional wellbeing and mental-health functioning	Mental-health-functioning measure	Structured study-level synthesis
K10	Psychological distress	Broad construct coverage	Non-specific psychological distress	Broad psychological distress measure	Structured study-level synthesis
CIDI	Diagnostic mood disorder	Diagnostic construct coverage	Structured diagnostic assessment of mood disorder	Structured diagnostic outcome	Descriptive synthesis
Self-reported depression/anxiety	Self-reported emotional problem	Limited construct specificity	Self-reported emotional complaint	Binary self-reported outcome	Descriptive synthesis

Instrument classifications represent interpretive coding decisions based on each instrument’s dominant intended construct, item content, and original purpose. They should not be interpreted as definitive claims that any instrument measures only one psychological construct or as a hierarchy of measurement validity.

### 2.7 Effect-size harmonization and statistical analysis

The included studies reported heterogeneous exposure definitions and contrasts, including blue-space visibility, proximity, coastal distance, water coverage, and area-based measures. Because several estimates represented changes per non-equivalent exposure units, converting regression coefficients by the outcome standard deviation alone would not create a common standardized mean difference.

Accordingly, no quantitative meta-analysis was performed. We extracted the original adjusted effect estimate, exposure contrast, outcome direction, covariate set, and outcome-measurement category for each study. Findings were synthesized using structured narrative comparison, descriptive evidence tables, and a study-level evidence map. Additional information on study-level analytic decisions and comparability judgments is provided in Supplementary Tables 2 and 4. Binary and structured diagnostic outcomes were summarized separately because they differed from continuous symptom and distress outcomes in ascertainment, effect-size metric, and interpretation. The structured narrative synthesis was informed by the Synthesis Without Meta-analysis (SWiM) reporting guideline to improve transparency in study grouping, presentation of study-level findings, and interpretation of synthesis limitations (Campbell et al., 2020).

### 2.8 Assessment of synthesis robustness and evidence limitations

Because no common interpretable exposure contrast, outcome construct, or effect metric was available, we did not conduct leave-one-out meta-analysis, formal subgroup tests, meta-regression, or formal assessment of reporting bias. Instead, we assessed the robustness of the structured synthesis by documenting the study design, exposure definition, exposure contrast, outcome construct, covariate adjustment, and estimate-selection decision for each study.

We also examined whether outcome-measurement categories co-occurred with other study-level characteristics, including participant age, country, exposure assessment method, study design, and covariate structure. This assessment was used to identify potential confounding between outcome-measurement category and other characteristics of the evidence base.

## 3 Results

### 3.1 Study selection and characteristics

**FIGURE 1:**
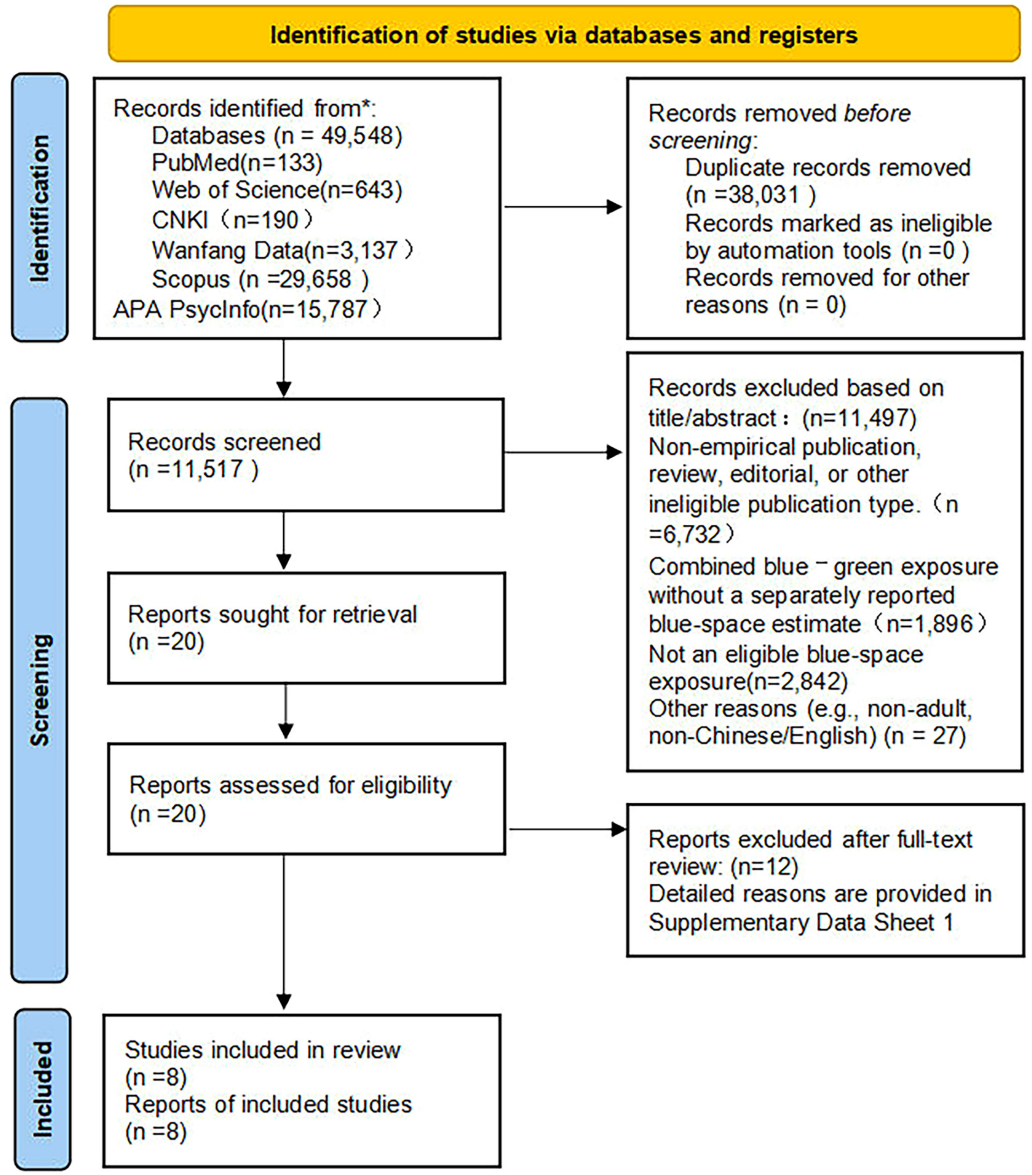
Preferred Reporting Items for Systematic Reviews and Meta-Analyses (PRISMA) 2020 flow diagram of the study selection process.

The database search identified 49,548 records. After duplicate removal, 11,517 records remained for title and abstract screening. At this stage, 11,497 records were excluded, leaving 20 reports for full-text assessment. All 20 full texts were retrieved and assessed for eligibility. Twelve reports were excluded after full-text review, and eight observational studies involving 35,797 adults were included in the review (Helbich et al., 2019; White et al., 2013; Liu et al., 2020, 2021; Chen and Yuan, 2020; Nutsford et al., 2016; Triguero-Mas et al., 2015; de Vries et al., 2016). The study-selection process is shown in Figure 1.

Four included studies were conducted in China, three in Europe, and one in New Zealand. Seven studies used cross-sectional designs, and one used a longitudinal design. Six studies reported continuous mental-health-related outcomes. Because their exposure definitions, exposure contrasts, and original effect metrics were not directly comparable, they were summarized using structured study-level evidence rather than a primary SMD synthesis. Two studies reported binary or structured diagnostic outcomes and were also summarized descriptively.

Methodological quality was generally high. Downs and Black checklist scores ranged from 13 to 16. These scores indicated generally complete reporting and coverage of the assessed checklist domains, but they did not eliminate concerns regarding residual confounding, exposure heterogeneity, or the limitations of predominantly cross-sectional evidence. Study characteristics, original exposure contrasts, adjusted effect estimates, covariate adjustment, and estimate-selection decisions are summarized in Table 2 and quality assessment scores are presented in Table 4.

**TABLE 4:** Quality assessment scores using the Downs and Black (1998) checklist (maximum score = 16).

No.	References	Study design	Confounding control	Exposure measurement	Outcome measurement	Results reporting	Statistical tests	Sample representativeness	Follow-up duration	Total
1	Helbich et al., 2019	2	2	2	2	2	2	1	0	13
2	White et al., 2013	2	2	2	2	2	2	2	2	16
3	Liu et al., 2021	2	2	2	2	2	2	1	0	13
4	Chen and Yuan, 2020	2	2	2	2	2	2	1	0	13
5	Nutsford et al., 2016	2	2	2	2	2	2	2	0	14
6	Liu et al., 2020	2	2	2	2	2	2	1	0	13
7	Triguero-Mas et al., 2015	2	2	1	2	2	2	2	0	13
8	de Vries et al., 2016	2	2	2	2	2	2	2	0	14

Scoring based on Downs and Black (1998) tool, total 16 points. Score ≥ 13, excellent; 10–12, good; 7–9, fair; ≤6 = poor.

### 3.2 Classification of depressive outcome instruments

The included studies assessed several related but non-equivalent mental-health constructs. Among the six studies reporting continuous outcomes, one used the GDS-15, which was classified as a depression-focused geriatric symptom measure (Helbich et al., 2019). The remaining five studies used broad psychological distress or mental-health-functioning measures, including the GHQ-12, MHI-5, SF-36 Mental Health subscale, and K10 (White et al., 2013; Liu et al., 2020, 2021; Chen and Yuan, 2020; Nutsford et al., 2016).

Two studies reported non-continuous outcomes. de Vries et al. (2016) used the CIDI to ascertain a structured diagnostic mood-disorder outcome, whereas Triguero-Mas et al. (2015) used a binary self-reported depression/anxiety item. These outcomes were retained in the descriptive synthesis because they differed from continuous symptom and distress measures in ascertainment, scale, and interpretation. Instrument classifications represent interpretive coding decisions based on dominant measurement purpose and content; they do not imply that any measure captures only one psychological construct. The interpretive classification of the outcome instruments is summarized in Table 3.

### 3.3 Study-level associations between blue space exposure and continuous mental-health-related outcomes

Six studies reported continuous mental-health-related outcomes. Their exposure definitions differed substantially, including street-view blue-space proportion, coastal proximity, water area within residential buffers, hydrophilicity indices, and visible blue-space coverage. The studies also differed in outcome construct, participant age, country, covariate adjustment, and analytic model. The original adjusted estimates, their exposure contrasts, and the covariate sets for the selected models are reported in Table 2.

**FIGURE 2:**
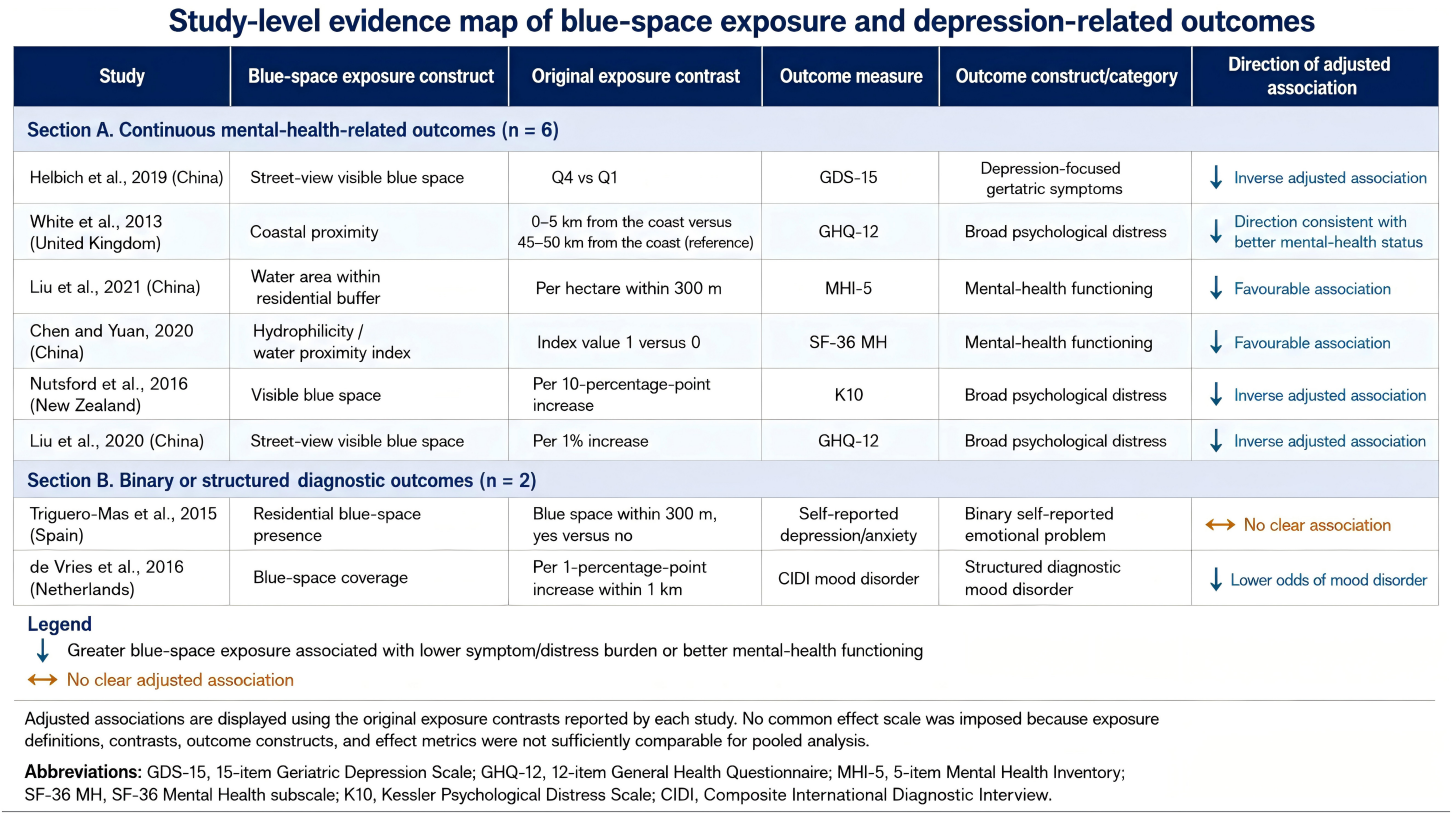
Study-level evidence map of blue-space exposure and depression-related outcomes. Adjusted associations are presented using the original exposure contrasts reported by each study. No common effect scale was imposed because blue-space exposure definitions, exposure contrasts, outcome constructs, and effect metrics were not sufficiently comparable for pooled analysis. Arrows indicate the direction of adjusted associations and do not imply causality. In White et al. (2013), the GHQ-12 outcome was reverse-scored; higher scores indicated lower psychological distress and better mental-health status. Positive coefficients therefore indicate a direction consistent with better mental-health status.

Across the six continuous-outcome studies, the reported adjusted associations were directionally compatible with either lower psychological distress or better mental-health functioning at greater blue-space exposure. However, the exposure contrasts, effect metrics, outcome scales, study populations, and covariate sets differed substantially across studies. Therefore, the magnitude of associations was not compared quantitatively across studies, and no pooled effect estimate was calculated. Figure 2 presents the study-level evidence map, including the original blue-space exposure contrasts, outcome constructs, and directions of adjusted associations across the eight included studies.

### 3.4 Distribution of outcome-measurement categories and study characteristics

Outcome-measurement categories were unequally represented. Only one study used a depression-focused geriatric symptom measure, whereas five studies used broad psychological distress or mental-health-functioning measures. The single GDS-15 study also differed from the remaining studies in participant age profile, geographical context, street-view-based exposure assessment, and covariate structure. Accordingly, the available evidence could not distinguish whether observed differences across studies reflected outcome measurement, exposure definition, population characteristics, residual confounding, or other study-level factors.

### 3.5 Binary and diagnostic outcomes

Two studies reported non-continuous depression-related outcomes and were summarized descriptively. de Vries et al. (2016), using a CIDI-based structured diagnostic mood-disorder outcome, reported that each 1% increase in blue-space coverage was associated with lower odds of mood disorder (OR = 0.97, 95% CI: 0.95 to 0.99). Triguero-Mas et al. (2015), using a binary self-reported depression/anxiety outcome, reported no clear association with blue space within 300 m (OR = 1.13, 95% CI: 0.90 to 1.41). Because these outcomes differed in ascertainment, exposure operationalization, and effect metric, they should not be interpreted as directly comparable with continuous depressive-symptom, psychological-distress, or mental-health-functioning outcomes.

### 3.6 Assessment of evidence robustness

Because the included studies differed in exposure constructs, exposure contrasts, outcome measures, study populations, and analytic models, assessment of robustness focused on transparent study-level comparison rather than on a pooled estimate. We documented alternative eligible estimates, original models, covariate adjustment, estimate-selection decisions, and study-level comparability judgments in Table 2 and Supplementary Tables 2–4.

The depression-focused outcome category contained only one study and co-occurred with an older population, a Chinese urban setting, and street-view-based exposure measurement. Therefore, the available evidence did not support a reliable inference that outcome-measurement category modified the association between blue-space exposure and depression-related outcomes.

The credibility of the evidence base was limited by predominantly cross-sectional designs, residual confounding, heterogeneous exposure definitions and contrasts, non-equivalent outcome constructs, and the small number of studies within each outcome-measurement category. Given the small number of studies and the absence of a common effect metric, we did not conduct a formal assessment of reporting bias or assign a formal certainty rating to a pooled association.

## 4 Discussion

### 4.1 Principal findings: from effect estimation to construct interpretation

The central finding of this review is not that outcome measurement has been shown to modify the association between blue space exposure and depression-related outcomes. Rather, the available evidence base is too sparse, outcome-heterogeneous, and imbalanced to test that question reliably. The included studies assessed related but non-equivalent constructs, including geriatric depressive symptoms, non-specific psychological distress, mental-health functioning, structured diagnostic mood disorder, and binary self-reported depression/anxiety.

One GDS-15-based study reported an inverse adjusted association between street-view blue-space exposure and geriatric depressive symptoms. However, its exposure contrast, outcome scale, participant population, geographical context, and analytic model differed from those used in the other studies. The finding therefore cannot be compared quantitatively in magnitude with estimates from studies using broader psychological distress or mental-health-functioning measures. The principal contribution of this review is therefore to identify an interpretive limitation in the current evidence base: outcome constructs should be specified more clearly when blue space–mental-health findings are compared or synthesized.

### 4.2 Outcome measurement as a source of construct-level heterogeneity

The present results align with a wider psychometric concern in depression research: depression is not a unitary outcome, and total-score-based approaches may obscure meaningful symptom-level heterogeneity. Fried and Nesse (2015b) argued that depression sum scores can mask differences among specific symptoms, while Fried (2017) demonstrated limited content overlap across widely used depression scales. From this perspective, pooling depression-related outcomes without regard to their psychometric content may introduce construct-level heterogeneity that conventional statistical heterogeneity indices cannot fully capture.

This issue is particularly consequential in environmental psychology and urban health research. Blue space exposure may plausibly influence affect regulation, perceived stress, cognitive restoration, rumination, loneliness, social participation, or behavioral activation. These pathways are psychologically meaningful but not equivalent. Theoretical accounts of nature and mental health emphasize restoration, stress reduction, attentional recovery, and affective regulation as plausible mechanisms linking natural environments to mental health (White et al., 2020; Kaplan, 1995; Bratman et al., 2019). Behavioral activation theory further suggests that environments supporting outdoor activity, routine engagement, and rewarding contextual contact may be relevant to mood-related processes (Jacobson et al., 2001). A measure emphasizing affective or cognitive depressive symptoms may therefore capture a different form of environmental responsiveness than a measure designed to screen for general distress or overall mental health functioning.

Broad psychological distress and mental-health-functioning measures should not be interpreted as invalid or irrelevant to depression-related research. The GHQ-12, K10, MHI-5, and SF-36 Mental Health subscale are widely used and clinically informative measures of psychological distress, emotional wellbeing, or broad mental-health status. Some of these measures have also been evaluated as screening tools for common mood and anxiety disorders in particular populations (Kessler et al., 2002; Friedman et al., 2005; Ten Have et al., 2024).

The issue is therefore not whether broad measures are legitimate mental-health outcomes. Rather, they differ from depression-focused symptom measures in construct specificity, item content, intended use, diagnostic purpose, and sensitivity to particular symptom domains. When studies using these different instruments are synthesized, the resulting estimate should be interpreted as an association with the measured construct rather than as a universal estimate of “depression” in a clinical or symptom-specific sense.

### 4.3 Why the GDS-15 study cannot be compared directly with broader outcome measures

The single GDS-15 study should not be interpreted as providing quantitatively comparable evidence relative to studies using broader psychological distress or mental-health-functioning measures, because the original exposure contrast, outcome scale, study population, and analytic model were not directly comparable. Although the GDS-15 is a depression-focused geriatric screening measure, the corresponding study also differed from the remaining evidence in participant age profile, Chinese urban context, street-view-based blue-space assessment, and potentially residual confounding (Helbich et al., 2019).

Outcome-measurement category was therefore inseparably confounded with several study-level characteristics. The present evidence cannot distinguish whether the observed study-level pattern reflects outcome specificity, population characteristics, cultural context, exposure definition, analytic decisions, chance, or an unmeasured combination of these factors. The finding is best understood as evidence that the field currently lacks enough studies using depression-focused measures to evaluate this question robustly.

### 4.4 Implications for blue space, environmental psychology, and depression research

The findings have implications beyond blue space exposure. Environmental mental health research has made substantial progress in refining exposure assessment, including distance-based metrics, land-cover measures, visibility indices, and street-view-derived environmental indicators (Helbich et al., 2019; Nutsford et al., 2016; Liu et al., 2020; Kwan, 2012). This progress is essential, but exposure precision alone is insufficient. A highly detailed exposure metric cannot compensate for an outcome measure that does not clearly correspond to the psychological construct of interest.

The blue space literature may therefore face a dual-measurement challenge. Researchers must determine whether blue space is best conceptualized as proximity, visibility, accessibility, use, contact, or perceived quality, while also determining whether the relevant psychological outcome is depressive symptom severity, non-specific distress, emotional wellbeing, mental health functioning, or clinical disorder. Existing blue space reviews have emphasized the potential mental health relevance of blue space exposure, but the present findings suggest that future syntheses should attend more closely to the psychometric meaning of the outcomes being pooled (White et al., 2020; Smith et al., 2021; Wang et al., 2025).

This point is especially relevant when interpreting the direction of study-level findings. Across the included continuous-outcome studies, several adjusted estimates were directionally compatible with lower psychological distress or better mental-health functioning at greater blue-space exposure. However, the exposure contrasts and outcome constructs were not sufficiently comparable to support a single pooled estimate or a quantitative comparison of effect magnitude. The available evidence therefore cannot determine whether differences across studies reflect weak environmental associations, exposure heterogeneity, outcome construct differences, residual confounding, or a combination of these factors.

The binary and diagnostic outcomes reinforce this concern. Diagnostic mood disorder assessed by CIDI and self-reported depression/anxiety represent different levels of psychological assessment. CIDI is a structured diagnostic interview designed to classify mental disorders rather than quantify continuous symptom burden (Kessler and Üstün, 2004; Haro et al., 2006). Diagnostic outcomes are threshold-based and may be less sensitive to subtle variation in subclinical symptoms. Self-reported depression/anxiety items may be vulnerable to construct ambiguity and reporting differences. Treating such outcomes as equivalent to continuous depressive symptom scales risks conflating symptom burden, psychological distress, self-identification, and clinical diagnosis.

### 4.5 Comparison with broader nature-based mental-health evidence

Previous reviews of blue space, green space, and broader nature-based mental-health research have generally reported potentially favorable associations with mental-health outcomes, while also emphasizing substantial heterogeneity in exposure definitions, populations, study designs, and outcome measures (Smith et al., 2021; Wang et al., 2025; Liu et al., 2023; Blakeslee et al., 2026). However, these evidence bases should not be treated as directly interchangeable. Residential exposure studies typically assess associations between ambient environmental conditions and mental-health outcomes, whereas nature-contact and green-exercise interventions assess more immediate, behaviorally mediated experiences of natural environments. Related evidence also suggests that different types of natural-environment exposure may correspond to different aspects of subjective well-being (White et al., 2017).

For example, recent meta-analytic evidence on green-space exposure has examined depression and anxiety outcomes using residential or area-based environmental indicators, whereas reviews of urban green exercise have synthesized intervention-based studies involving active engagement with green environments (Liu et al., 2023; Hu et al., 2025). These differences represent distinct exposure constructs and causal contrasts. Therefore, the small or inconsistent associations observed in blue-space research should not be interpreted as necessarily weaker than effects reported for green-space interventions or other nature-contact studies. Instead, comparisons should account for whether the evidence concerns residential exposure, visibility, access, use, or structured intervention.

### 4.6 A methodological agenda for future research

Future blue space–mental-health studies should select outcome measures according to the construct they intend to examine rather than applying a single instrument as a universal standard. When the research question concerns depressive symptom burden, a depression-focused measure such as the PHQ-9, CES-D, or HADS-D may be appropriate. When the hypothesized pathway concerns non-specific distress, emotional wellbeing, or broader mental-health functioning, broader instruments may be more suitable (Radloff, 1977; Zigmond and Snaith, 1983; Kroenke et al., 2001).

Outcome selection should be aligned with the proposed mechanism linking blue space exposure to mental health. Studies focused on cognitive restoration may assess rumination, attentional fatigue, or cognitive symptoms; studies focused on stress recovery may assess perceived stress, affective distress, or physiological stress indicators; and studies focused on behavioral activation or social pathways may assess outdoor activity, social participation, anhedonia, and engagement with rewarding activities (Kaplan, 1995; Bratman et al., 2019; Jacobson et al., 2001). Where feasible, researchers should retain item-level or domain-level outcome data to improve interpretability, while recognizing that the most suitable level of detail depends on the study’s theoretical objective and population.

This agenda implies several design priorities. Studies should prespecify whether depression-related outcomes are treated as depression-specific symptoms, general distress, wellbeing, functioning, or diagnostic disorder. Item-level or domain-level symptom data should be retained rather than reduced exclusively to total scores. Exposure assessment should distinguish residential proximity, visual exposure, perceived access, and actual use of blue spaces. Longitudinal designs are needed to determine whether blue space exposure predicts changes in symptom domains over time rather than merely correlating with cross-sectional differences. Future meta-analyses should code outcome-measurement paradigm as a prespecified moderator instead of using it only as a *post hoc* explanation for heterogeneity.

Such an approach would move the field beyond generic claims that blue space is “good for mental health” toward more precise, testable propositions about which forms of blue space exposure are associated with which components of depression, for whom, under what conditions, and through which mechanisms.

### 4.7 Strengths and limitations

This study reframes the blue space–depression evidence base through a psychometric lens and addresses an underexamined source of heterogeneity in environmental mental health research. It distinguishes depression-specific symptom scales, general distress and mental health functioning instruments, and binary or diagnostic outcomes, rather than treating all depression-related outcomes as equivalent. It also combines structured study-level evidence synthesis with an explicit outcome-measurement classification framework, allowing findings to be interpreted in relation to construct coverage rather than statistical significance alone.

Several limitations require emphasis. Only six studies reported continuous outcomes, and only one used a depression-focused symptom measure. Consequently, the evidence was insufficient to test whether outcome-measurement category modified the association between blue space exposure and mental-health outcomes. Outcome category was also confounded with study-level characteristics, including participant age, geographical context, exposure assessment method, and potentially residual confounding.

In addition, the included exposure contrasts were heterogeneous, including visibility, proximity, water coverage, and area-based measures. These contrasts cannot automatically be transformed into one common, interpretable causal effect estimate. Most studies were cross-sectional, limiting causal inference, and publication bias could not be assessed reliably because the number of studies was small. Accordingly, this review should be interpreted as a construct-focused appraisal of the evidence base rather than as definitive evidence that blue space exposure has different effects according to outcome-measurement category. Rather than assigning a formal certainty rating to a pooled effect, this review evaluated the limitations of the evidence base through transparent reporting of study design, exposure contrast, covariate adjustment, outcome construct, and estimate-selection decisions.

## 5 Conclusion

This systematic review shows that blue space studies have assessed several related but non-equivalent outcomes, including depression-focused symptoms, broad psychological distress, mental-health functioning, structured diagnostic outcomes, and binary self-reported emotional problems. The current evidence base is too sparse, heterogeneous, and imbalanced to determine whether outcome-measurement category reliably changes the association between blue space exposure and depression-related outcomes.

The single GDS-15-based study should not be interpreted as evidence of symptom-dimensional specificity or as evidence that depression-focused measures yield stronger blue-space associations than broader measures. The main implication is not that all future studies should use one standardized instrument, but that researchers should define their intended mental-health construct clearly, select outcomes that align with their theoretical mechanisms and populations, and report sufficient construct-level detail for transparent evidence synthesis.

More longitudinal and well-characterized studies using clearly specified exposure contrasts and theory-aligned outcome measures are needed before the field can determine whether particular forms of blue space exposure are associated with particular depression-related symptom domains.

## Supplementary material

Supplementary Table 1 Complete database-specific search strategies.

Supplementary Table 2 Study-level analytic decisions and candidate exposure estimates.

Supplementary Table 3 Alternative eligible estimates, exposure buffers, original models, and estimate-selection decisions.

Supplementary Table 4 Study-level analytic comparability assessment.
